# Research on Similarity Measurement for Texture Image Retrieval

**DOI:** 10.1371/journal.pone.0045302

**Published:** 2012-09-25

**Authors:** Zhengli Zhu, Chunxia Zhao, Yingkun Hou

**Affiliations:** 1 School of Computer Science and Technology, Nanjing University of Science and Technology, Nanjing, China; 2 College of Information Science and Technology, Nanjing Forestry University, Nanjing, China; 3 School of Information Science and Technology, Taishan University, Taian, China; Medical University of Graz, Austria

## Abstract

A complete texture image retrieval system includes two techniques: texture feature extraction and similarity measurement. Specifically, similarity measurement is a key problem for texture image retrieval study. In this paper, we present an effective similarity measurement formula. The MIT vision texture database, the Brodatz texture database, and the Outex texture database were used to verify the retrieval performance of the proposed similarity measurement method. Dual-tree complex wavelet transform and nonsubsampled contourlet transform were used to extract texture features. Experimental results show that the proposed similarity measurement method achieves better retrieval performance than some existing similarity measurement methods.

## Introduction

With the rapid expansion of digital image libraries and multimedia databases, content-based image retrieval (CBIR) has become a hot research topic in the computer science field. Content of images includes color characteristics, shape characteristics, texture characteristics, and semantics characteristics. Because texture is a type of inherent property for most physical surfaces, texture characteristics usually play an important role in CBIR.

A complete texture image retrieval system includes two techniques: texture features extraction and similarity measurement. Texture features used in CBIR are usually extracted by space-frequency domain approaches [1]–[8]. Shutao Li and John Shawe-Taylor used a wavelet transform and a contourlet transform to extract texture features for image classifying [9]. A Gabor filter has a nice effect on descripting texture features, but it still has two shortcomings: one is that redundant information is produced after different Gabor filters; the other is that feature extraction by Gabor filters usually has considerably high computational complexity. N. Kingsbury et al used a dual-tree complex wavelet transform (DT-CWT) to extract texture features [10]–[12]. DT-CWT can overcome two drawbacks of the Discrete Wavelet Transform (DWT); one is that invariance, the other is that DWT has only limited directivity. DT-CWT not only has good localization in time-frequency domains, but also has approximate translation invariance, more directivity and limited data redundancy. A nonsubsampled contourlet transform (NSCT) has anisotropy and translation invariance [13]–[14]. In this paper, DT-CWT and NSCT are respectively used to extract texture features of images.

Similarity measurement is a key technique for texture image retrieval. Kokare. M. et al in [15] compared nine distance similarity measurements, such as Weighted-Mean-Variance distance (WMVD), Euclidean distance (ED), Canberra distance (CD), Bray-Curtis distance (BCD), Manhattan distance, Mahalanobis distance, Chebyshev distance, Squared Chi-Squared distance, and Squared Chord distance for texture image retrieval. Experimental results show that WMVD, CD and BCD are the three best distance similarity measurements for image retrieval problems, but the retrieval rates are not ideal when using WMVD, CD and BCD. Therefore, exploring more effective similarity measurements is a problem worth studying.

In this paper, we present an effective similarity measurement. A dual-tree complex wavelet transform and a nonsubsampled contourlet transform were respectively used to extract texture features. The MIT vision texture database (640 images), the Brodatz texture database (1776 images), and the Outex texture database (5104 images) were used to verify the retrieval performance. Experimental results show that the retrieval performance can be improved by the proposed similarity measurement more than some existing similarity distance measurement methods.

## Methods

### 2.1 Related Works

Because WMVD, CD and BCD are the best three similarity measurements for image retrieval [15], so here we focus on the WMVD similarity measurement, the CD similarity measurement and the BCD similarity measurement.

WMVD (Weighted-Mean-Variance distance) is widely used in image retrieval [1] [7]. Generally, two patterns 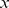 and 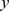 are considered, where 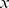 is a query image and 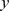 is a target image in the database. 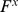
and 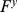 are, respectively, the feature vectors of 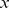 and 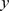. The WMV is defined as the following,
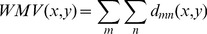
(1)


Where
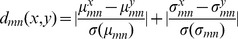
Where 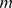 denotes the scale, and 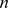 is the number of subbands in each scale.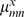 and 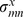 are the mean and the standard deviation of each subband for a query image. 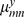 and 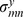 are the mean and the standard deviation of each subband for a target image. 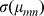 and 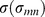 are the standard deviations of 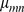 and 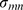 respectively over the entire database, and they are used to normalize the individual feature components.

If 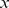 and 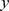 are two n-dimensional feature vectors of an image to retrieve and query the image:

The ED is defined as the following,
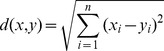
(2)the CD is defined as the following,
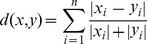
(3)and the BCD is defined as the following,



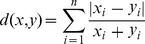
(4)In this paper, we present an effective distance measurement that is more effective than the above three distance measurement methods.

**Figure 1:**
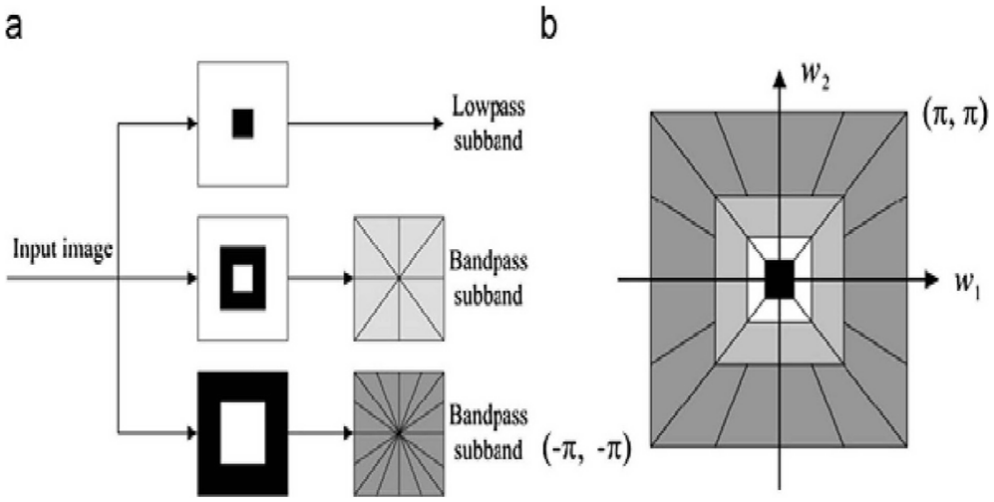
Two-level nonsubsampled contourlet transform decomposition. (a) NSFB structure that implements the NSCT (b) The obtained Frequency partitioning.

**Figure 2:**
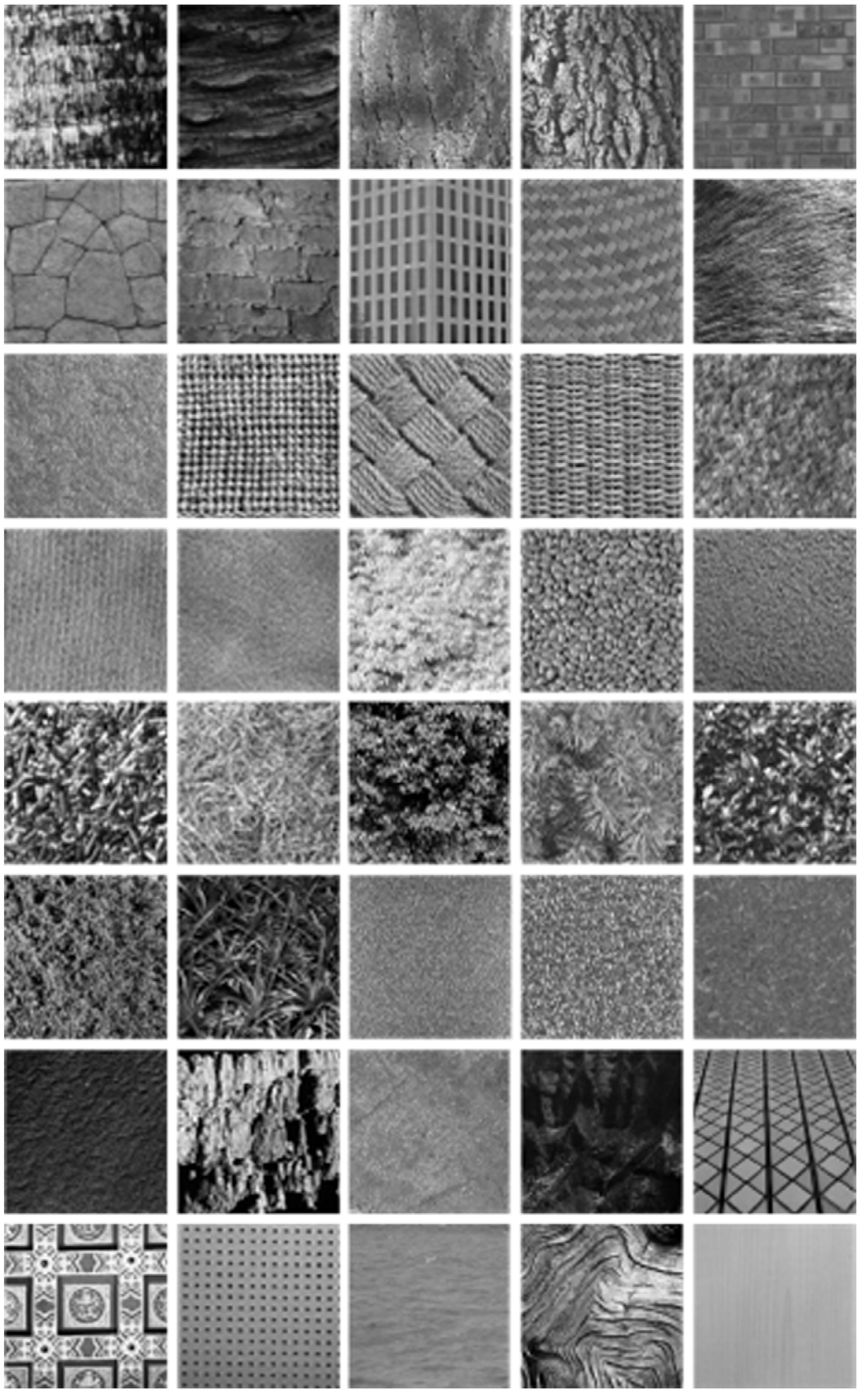
40 different classes of texture images from the MIT texture database.

**Figure 3:**
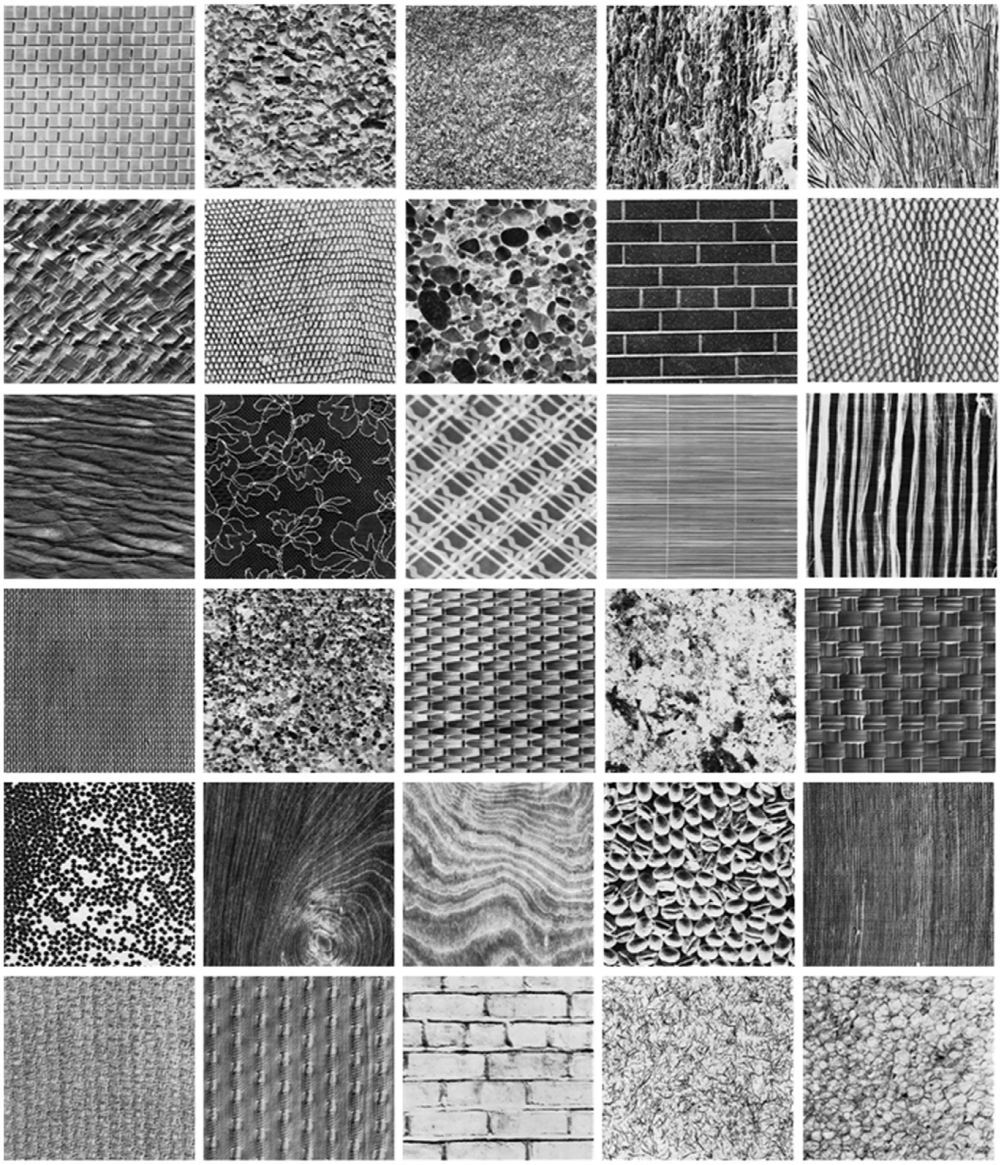
Different classes of texture images from the Brodatz texture database.

**Figure 4:**
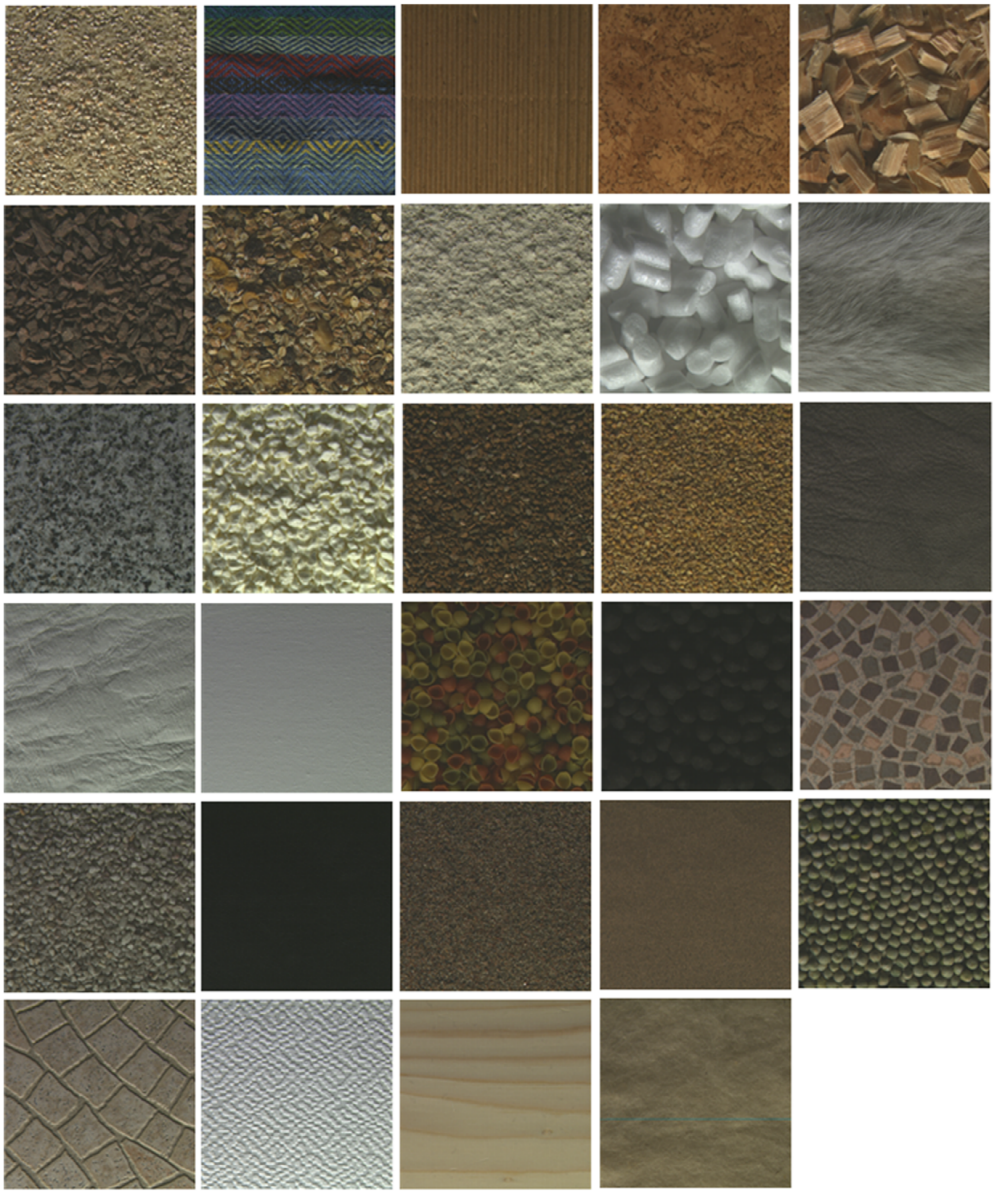
One example from each category in the Outex texture database.

### 2.2 Proposed Distance Similarity Measure

We present an effective Average Euclidean distance (AED). If X and Y are two n-dimensional feature vectors of an image from a database and the query image, we give the new similarity measurement as the following,
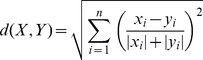
(5)


The proposed distance similarity measurement not only contains some relations between objects, but also comprehensively considers all dimensional feature parameters. When the proposed similarity distance measurement is used for texture image retrieval, the experimental results show that the retrieval performances are better using the proposed similarity distance measurement than the existing similarity measurements.

**Table 1:** Comparison of retrieval performance of different types of metrics for texture image retrieval using NSCT on the MIT image database (640 images of 40 different classes).

*Texture name*	*ED*	*WMVD*	*CD*	*BCD*	*Proposed method*
Bark 0	22.27	26.18	21.10	21.10	23.05
Bark 6	62.89	58.99	57.82	57.82	61.33
Bark 8	39.06	42.58	35.16	35.16	45.32
Bark 9	30.86	31.25	26.96	26.96	30.08
Brick 1	92.58	99.22	100.0	100.0	100.0
Brick 4	74.61	79.69	89.85	89.85	89.46
Brick 5	46.48	66.80	57.43	57.43	67.19
Buildings 9	80.47	82.43	88.68	88.68	92.19
Fabric 0	83.98	81.64	91.02	91.02	85.94
Fabric 4	46.09	50.00	39.46	39.46	53.52
Fabric 7	71.48	76.18	76.96	76.96	87.11
Fabric 9	100.0	99.61	96.88	96.88	98.83
Fabric 11	73.44	79.30	77.35	77.35	86.33
Fabric 14	99.61	99.22	98.44	98.44	99.22
Fabric 15	67.58	72.66	76.96	76.96	86.33
Fabric 17	98.83	99.61	94.54	94.54	95.32
Fabric 18	89.06	92.58	90.63	90.63	95.71
Flowers 5	89.45	86.72	98.05	98.05	98.83
Food 0	97.27	98.05	92.58	92.58	100.0
Food 5	60.94	61.72	60.55	60.55	70.71
Food 8	69.92	80.08	66.41	66.41	78.13
Grass 1	67.97	81.64	75.39	75.39	82.43
Leaves 8	63.67	64.45	57.04	57.04	60.94
Leaves 10	49.22	55.47	40.24	40.24	47.27
Leaves 11	67.58	67.19	61.72	61.72	67.58
Leaves 12	76.95	67.58	64.07	64.07	72.27
Leaves 16	65.63	55.08	60.94	60.94	73.44
Metal 0	78.91	85.16	92.19	92.19	92.58
Metal 2	97.27	98.05	99.22	99.22	100.0
Misc 2	99.22	96.88	98.05	98.05	98.83
Sand 0	96.09	99.22	95.32	95.32	97.66
Stone 1	33.20	46.88	30.86	30.86	37.11
Stone 4	83.98	91.02	91.41	91.41	94.54
Terrain 10	58.59	55.47	57.04	57.04	57.04
Title 1	58.59	47.27	53.91	53.91	52.35
Title 4	86.72	91.41	89.46	89.46	94.14
Title 7	58.98	67.97	66.41	66.41	80.86
Water 5	100.0	100.0	100.0	100.0	99.61
Wood 1	29.30	37.11	39.46	39.46	41.02
Wood 2	100.0	100.0	100.0	100.0	100.0
**Average**	**71.72%**	**74.31%**	**72.74%**	**72.74%**	**77.36%**

**Table 2:** Comparison of retrieval performance of different types of metrics for texture image retrieval using DT-CWT on the MIT image database (640 images of 40 different classes).

*Texture name*	*ED*	*WMVD*	*CD*	*BCD*	*Proposed method*
Bark 0	27.34	37.11	38.28	38.28	43.36
Bark 6	60.55	61.33	55.47	55.47	58.98
Bark 8	41.41	43.75	42.97	42.97	46.88
Bark 9	30.86	30.47	33.20	33.20	35.16
Brick 1	93.36	100.0	100.0	100.0	100.0
Brick 4	77.34	90.23	91.41	91.41	89.84
Brick 5	47.27	85.94	89.45	89.45	90.63
Buildings 9	83.59	98.05	96.09	96.09	95.70
Fabric 0	91.80	85.94	86.72	86.72	86.72
Fabric 4	54.30	69.92	83.60	83.60	81.25
Fabric 7	83.2	90.63	90.63	90.63	93.75
Fabric 9	100.0	99.22	99.22	99.22	99.22
Fabric 11	79.69	70.31	71.88	71.88	71.09
Fabric 14	95.31	100.0	100.0	100.0	100.0
Fabric 15	74.61	88.67	87.89	87.89	90.23
Fabric 17	100.0	100.0	100.0	100.0	100.0
Fabric 18	88.67	98.44	98.83	98.83	99.61
Flowers 5	88.28	92.97	87.11	87.11	91.80
Food 0	88.28	93.75	93.75	93.75	96.09
Food 5	71.88	58.20	58.20	58.20	64.06
Food 8	63.67	87.89	85.94	85.94	86.72
Grass 1	71.48	83.20	87.50	87.50	87.50
Leaves 8	69.14	79.69	82.03	82.03	82.81
Leaves 10	35.94	51.56	51.17	51.17	53.13
Leaves 11	63.67	73.44	71.48	71.48	75.78
Leaves 12	78.52	80.08	82.42	82.42	84.38
Leaves 16	63.67	73.05	68.36	68.36	75.39
Metal 0	67.97	89.45	87.11	87.11	85.16
Metal 2	99.22	100.0	100.0	100.0	100.0
Misc 2	98.44	99.22	99.61	99.61	99.61
Sand 0	92.19	98.44	97.66	97.66	99.22
Stone 1	48.44	80.47	78.91	78.91	80.47
Stone 4	81.64	91.80	90.63	90.63	92.19
Terrain 10	56.64	50.00	43.75	43.75	47.27
Title 1	57.81	58.98	57.81	57.81	55.86
Title 4	92.58	96.88	97.27	97.27	98.44
Title 7	62.11	80.47	83.20	83.20	88.67
Water 5	100.0	100.0	98.44	98.44	98.44
Wood 1	15.63	40.63	49.22	49.22	50.78
Wood 2	100.0	100.0	100.0	100.0	100.0
**Average**	**72.41%**	**80.25%**	**80.43%**	**80.43%**	**81.91%**

**Table 3:** Comparison of retrieval performance of different types of metrics for texture image retrieval using NSCT on the Brodatz image database (1776 images of 111 different classes).

*Texture name*	*ED*	*WMVD*	*CD*	*BCD*	*Proposed method*
D1	79.69	88.28	75.39	75.39	87.89
D2	42.19	54.30	38.28	38.28	44.53
D3	75.00	76.95	76.17	76.17	85.16
D4	67.97	79.30	78.52	78.52	91.02
D5	69.92	54.30	62.50	62.50	66.41
D6	92.58	86.72	94.92	94.92	98.44
D7	28.91	38.28	25.39	25.39	31.64
D8	53.91	95.70	67.19	67.19	78.52
D9	66.02	48.83	57.42	57.42	69.53
D10	75.78	46.88	80.47	80.47	87.11
D11	77.73	71.88	73.83	73.83	81.64
D12	51.95	68.75	50.00	50.00	56.25
D13	37.50	37.50	31.25	31.25	41.02
D15	85.94	83.20	76.56	76.56	82.42
D16	97.66	98.44	96.09	96.09	100.0
D17	86.72	85.55	66.80	66.80	91.02
D18	53.52	56.64	62.89	62.89	78.52
D19	76.17	79.30	73.44	73.44	82.42
D20	99.61	100.0	100.0	100.0	100.0
D21	100.0	100.0	100.0	100.0	100.0
D22	66.41	48.44	62.50	62.50	71.09
D23	35.16	44.92	26.17	26.17	32.42
D24	73.05	80.47	60.16	60.16	69.92
D25	87.89	56.25	79.30	79.30	88.28
D26	96.09	90.23	91.41	91.41	96.88
D27	39.06	56.25	41.02	41.02	48.83
D28	70.31	85.16	64.84	64.84	76.56
D29	89.45	87.11	74.61	74.61	86.33
D30	27.73	41.80	32.81	32.81	37.89
D31	31.25	29.69	21.09	21.09	21.88
D32	96.88	100.0	84.38	84.38	92.58
D33	92.58	89.06	78.91	78.91	89.84
D34	92.19	82.03	67.97	67.97	95.31
D35	94.92	96.49	86.33	86.33	94.92
D36	57.81	57.03	47.27	47.27	69.14
D37	59.77	43.75	66.41	66.41	76.95
D38	37.50	57.81	34.77	34.77	47.27
D39	48.44	32.81	37.50	37.50	44.53
D40	61.72	37.50	48.05	48.05	55.47
D41	64.06	41.41	64.45	64.45	70.31
D42	45.31	43.36	34.77	34.77	41.80
D43	16.80	15.24	19.53	19.53	19.14
D44	28.52	16.02	19.53	19.53	18.36
D45	35.94	12.89	34.38	34.38	26.56
D46	99.61	99.22	97.66	97.66	98.05
D47	98.05	98.83	100.0	100.0	99.61
D48	69.53	97.27	92.97	92.97	94.92
D49	100.0	100.0	100.0	100.0	100.0
D50	44.14	36.33	41.02	41.02	48.83
D51	63.67	83.99	50.00	48.05	46.49
D52	51.17	40.63	44.14	44.14	54.69
D53	99.61	100.0	97.27	97.27	100.0
D54	43.36	38.67	36.72	36.72	38.28
D55	85.55	98.83	76.95	76.95	89.06
D56	80.47	100.0	88.67	88.67	96.49
D57	100.0	100.0	91.80	91.80	99.22
D58	14.45	17.19	16.80	16.80	17.97
D59	23.05	26.95	31.25	31.25	32.81
D60	35.55	34.38	39.84	39.84	42.58
D61	35.16	34.38	32.81	32.81	42.19
D62	30.08	45.70	26.17	26.17	28.52
D63	56.64	32.81	64.45	64.45	69.14
D64	76.95	65.24	78.52	78.52	85.94
D65	87.89	98.44	74.61	74.61	82.81
D66	79.30	57.81	63.67	63.67	76.56
D67	44.53	57.42	35.94	35.94	39.84
D68	70.31	88.28	75.00	75.00	85.55
D69	47.27	60.16	49.22	49.22	57.03
D70	40.63	39.45	45.31	45.31	49.22
D71	65.23	84.38	71.88	71.88	81.64
D72	22.66	36.72	30.86	30.86	45.31
D73	30.08	36.72	25.39	25.39	32.03
D74	87.89	79.30	73.83	73.83	82.42
D75	96.48	93.36	99.22	99.22	99.61
D76	68.75	69.53	64.45	64.45	69.92
D77	88.67	100.0	82.42	82.42	96.09
D78	80.47	76.17	76.17	76.17	80.47
D79	64.45	76.56	64.45	64.45	69.92
D80	71.09	75.39	69.14	69.14	78.91
D81	48.05	69.92	41.02	41.02	52.34
D82	77.34	88.28	77.34	77.34	82.42
D83	96.88	99.61	93.36	93.36	97.66
D84	96.48	98.44	91.80	91.80	96.49
D85	50.39	82.42	52.74	52.74	57.03
D86	68.36	48.44	66.80	66.80	72.27
D87	88.28	89.06	72.66	72.66	78.91
D88	35.16	48.44	26.95	26.95	29.69
D89	42.19	26.17	50.39	50.39	55.08
D90	21.09	34.77	23.83	23.83	27.34
D91	22.27	33.20	29.30	29.30	29.30
D92	98.83	97.27	97.66	97.66	100.0
D93	48.44	84.77	60.94	60.94	78.91
D94	49.61	44.53	54.69	54.69	65.63
D95	84.77	91.02	71.49	71.49	85.94
D96	71.09	46.09	81.25	81.25	83.99
D97	50.78	31.64	44.14	44.14	46.88
D98	67.19	53.13	61.72	61.72	71.09
D99	42.97	32.81	41.41	41.41	45.70
D100	33.59	35.16	26.56	26.56	39.84
D101	99.22	52.74	98.83	98.83	94.14
D102	100.0	61.33	98.44	95.70	80.08
D103	90.63	59.38	89.45	89.45	89.06
D104	92.19	51.95	91.41	91.41	89.84
D105	78.91	63.67	78.52	78.52	78.13
D106	71.88	64.06	63.28	63.28	74.22
D107	73.44	67.58	55.08	55.08	66.02
D108	54.30	32.03	51.95	51.95	54.30
D109	71.48	56.64	71.49	71.49	76.56
D110	87.11	57.42	94.14	94.14	97.66
D111	68.75	71.88	57.03	57.03	70.31
D112	41.41	41.02	45.70	45.70	52.34
**Average**	**65.26%**	**63.89%**	**62.48%**	**62.44%**	**68.98%**

**Table 4:** Comparison of retrieval performance of different types of metrics for texture image retrieval using DT-CWT on the Brodatz image database (1776 images of 111 different classes).

*Texture name*	*ED*	*WMVD*	*CD*	*BCD*	*Proposed method*
D1	98.44	99.22	97.27	97.27	96.88
D2	46.09	69.14	82.42	82.42	85.16
D3	83.98	83.59	89.84	89.84	91.80
D4	90.63	91.41	99.61	99.61	99.22
D5	74.61	71.88	59.38	59.38	61.72
D6	100.0	99.61	100.0	100.0	100.0
D7	35.55	50.39	50.39	50.39	53.13
D8	53.13	87.11	97.66	97.66	98.83
D9	74.22	91.41	92.19	92.19	95.31
D10	80.08	83.59	82.03	82.03	84.77
D11	90.63	96.48	93.75	93.75	96.09
D12	63.28	78.91	78.52	78.52	78.91
D13	48.44	62.50	51.17	51.17	52.73
D15	81.25	81.25	81.25	81.25	80.08
D16	99.22	100.0	100.0	100.0	100.0
D17	100.0	100.0	100.0	100.0	100.0
D18	81.25	87.89	90.23	90.23	94.92
D19	82.81	92.97	91.02	91.02	94.92
D20	100.0	100.0	100.0	100.0	100.0
D21	100.0	100.0	100.0	100.0	100.0
D22	76.17	79.69	71.88	71.88	74.61
D23	43.36	50.39	42.58	42.58	45.31
D24	85.94	89.45	88.28	88.28	91.80
D25	91.80	94.53	97.27	97.27	98.44
D26	100.0	100.0	98.05	98.05	98.83
D27	47.66	60.94	67.19	67.19	69.53
D28	69.14	88.67	93.36	93.36	94.14
D29	89.84	96.48	94.14	94.14	97.66
D30	27.34	33.98	47.27	47.27	43.36
D31	29.69	30.47	25.78	25.78	28.13
D32	99.22	100.0	100.0	100.0	100.0
D33	91.80	92.19	94.53	94.53	96.49
D34	100.0	98.44	99.22	99.22	100.0
D35	83.98	93.36	89.84	89.84	87.11
D36	65.63	67.58	62.50	62.50	62.50
D37	92.58	98.83	97.27	97.27	99.22
D38	39.06	62.89	76.17	76.17	78.13
D39	50.78	48.44	42.97	42.97	48.83
D40	58.20	62.50	62.50	62.50	67.58
D41	69.14	74.61	73.44	73.44	78.91
D42	40.23	47.27	49.61	49.61	53.91
D43	17.97	19.53	17.58	17.58	16.41
D44	33.59	32.03	22.27	22.27	19.92
D45	25.39	26.56	19.53	19.53	17.97
D46	96.09	97.66	94.92	94.92	91.41
D47	100.0	100.0	100.0	100.0	100.0
D48	67.19	94.53	89.06	89.06	90.24
D49	100.0	100.0	82.42	82.42	73.05
D50	57.42	61.72	71.48	71.48	75.00
D51	79.30	93.36	85.55	85.55	85.16
D52	62.50	54.30	53.52	53.52	60.55
D53	100.0	100.0	100.0	100.0	100.0
D54	54.30	57.81	49.61	49.61	50.78
D55	100.0	100.0	100.0	100.0	100.0
D56	94.53	100.0	100.0	100.0	100.0
D57	100.0	100.0	100.0	100.0	100.0
D58	15.63	19.14	18.75	18.75	20.70
D59	23.44	29.30	26.95	26.95	30.08
D60	38.67	52.34	60.55	60.55	61.72
D61	36.33	45.70	43.75	43.75	49.22
D62	29.69	46.48	51.56	51.56	51.56
D63	55.86	59.38	51.17	51.17	55.47
D64	86.72	87.89	96.09	96.09	96.88
D65	98.44	100.0	100.0	100.0	100.0
D66	91.41	92.97	96.48	96.48	100.0
D67	49.61	60.94	72.27	72.27	68.36
D68	88.28	97.66	90.63	90.63	94.14
D69	39.84	46.48	48.83	48.83	48.44
D70	46.09	48.05	52.73	52.73	56.25
D71	59.77	83.98	95.70	95.70	94.14
D72	41.80	55.47	51.56	51.56	55.47
D73	32.03	39.84	42.97	42.97	45.70
D74	74.22	85.94	86.33	86.33	85.16
D75	91.80	94.53	99.61	99.61	99.61
D76	96.09	98.44	94.53	94.53	97.66
D77	96.09	100.0	100.0	100.0	100.0
D78	87.89	86.33	90.23	90.23	92.19
D79	76.95	89.84	91.80	91.80	94.53
D80	82.42	91.02	94.92	94.92	98.83
D81	71.48	95.31	98.44	98.44	99.61
D82	98.05	100.0	100.0	100.0	100.0
D83	100.0	100.0	100.0	100.0	100.0
D84	96.88	99.61	100.0	100.0	100.0
D85	77.73	94.53	97.66	97.66	99.22
D86	77.73	82.42	86.72	86.72	90.24
D87	87.50	95.31	96.09	96.09	96.88
D88	32.81	41.41	38.67	38.67	39.06
D89	38.28	38.67	33.20	33.20	37.50
D90	22.66	35.55	59.77	59.77	61.72
D91	23.44	28.52	42.97	42.97	43.75
D92	87.89	97.66	100.0	100.0	100.0
D93	58.59	86.33	92.58	92.58	91.80
D94	67.97	78.13	73.05	73.05	78.52
D95	98.44	96.09	99.61	99.61	100.0
D96	97.66	97.27	87.89	87.89	91.80
D97	36.72	47.66	54.69	54.69	50.78
D98	67.19	70.31	64.84	64.84	66.41
D99	44.92	45.31	41.02	41.02	41.80
D100	42.97	43.75	40.63	40.63	45.70
D101	97.27	94.14	87.89	87.89	94.92
D102	99.61	100.0	92.19	92.19	100.0
D103	77.73	73.05	76.95	76.95	76.95
D104	77.73	62.89	65.23	65.23	61.72
D105	77.73	69.92	62.89	62.89	60.16
D106	67.97	63.67	65.63	65.63	67.58
D107	66.02	77.34	76.17	76.17	78.52
D108	54.30	60.55	51.56	51.56	53.13
D109	80.08	82.42	81.64	81.64	86.72
D110	92.19	98.05	98.05	98.05	100.0
D111	71.09	84.38	87.11	87.11	89.06
D112	48.05	59.77	57.81	57.81	60.55
**Average**	**70.28%**	**76.10%**	**76.26%**	**76.26%**	**77.66%**

**Table 5:** Comparison of retrieval performance of different types of metrics for texture image retrieval using NSCT on the Outex image database (5104 images of 319 different classes).

*Texture name*	*ED*	*WMVD*	*CD*	*BCD*	*Proposed method*
Barleyrice(11)	30.01	27.52	30.36	30.36	32.99
Canvas(46)	50.44	43.52	53.30	53.30	58.69
Cardboard(1)	38.67	31.25	52.74	52.74	58.59
Carpet(12)	23.18	28.42	39.94	39.94	46.29
Chips(23)	28.92	15.03	27.14	27.14	27.53
Crushedstone(8)	47.66	44.19	57.42	57.42	61.57
Flakes(10)	44.26	30.31	41.92	41.92	46.56
Flour(13)	39.03	37.05	48.50	48.50	52.74
Foam(4)	35.65	31.84	38.09	38.09	46.29
Fur(12)	53.48	44.73	53.74	53.74	55.37
Granite(10)	46.92	30.31	44.84	44.84	49.22
Granular(3)	54.95	50.65	65.37	65.37	72.79
Gravel(7)	43.53	36.38	50.78	50.78	53.35
Groats(7)	43.64	38.34	52.12	52.12	58.76
Leather(5)	50.31	54.53	59.22	59.22	61.09
Mineral(6)	41.15	41.47	53.91	53.91	59.51
Paper(10)	59.42	71.02	81.25	81.25	83.71
Pasta(6)	50.33	38.41	55.60	55.60	59.05
Pellet(4)	48.34	46.78	47.85	47.85	46.88
Plastic(47)	33.14	33.27	45.69	45.69	50.01
Quartz(6)	41.08	36.98	44.27	44.27	50.20
Rubber(1)	82.81	68.36	90.24	90.24	96.49
Sand(5)	45.55	35.47	48.83	48.83	51.41
Sandpaper(8)	39.55	34.43	46.39	46.39	47.12
Seeds(13)	60.94	48.08	57.42	57.42	63.70
Tile(7)	26.51	23.05	38.28	38.28	40.96
Wallpaper(20)	37.50	56.43	45.00	45.00	57.56
Wood(12)	35.09	53.13	57.75	57.75	64.68
Wool(2)	41.21	44.15	57.62	57.62	60.94
**Average**	**41.51%**	**38.87%**	**48.31%**	**48.31%**	**52.93%**

**Table 6:** Comparison of retrieval performance of different types of metrics for texture image retrieval using DT-CWT on the Outex image database (5104 images of 319 different classes).

*Texture name*	*ED*	*WMVD*	*CD*	*BCD*	*Proposed method*
Barleyrice(11)	31.64	29.94	29.23	29.23	30.58
Canvas(46)	59.65	57.19	58.96	58.96	61.54
Cardboard(1)	73.83	73.05	72.66	72.66	83.59
Carpet(12)	30.99	49.09	46.32	46.32	48.28
Chips(23)	25.24	20.69	20.41	20.41	19.91
Crushedstone(8)	50.93	60.26	60.30	60.30	62.11
Flakes(10)	42.54	40.16	39.57	39.57	40.04
Flour(13)	42.88	54.00	55.23	55.23	57.36
Foam(4)	48.24	51.47	46.49	46.49	49.71
Fur(12)	53.65	53.42	49.87	49.87	49.91
Granite(10)	54.03	42.27	40.98	40.98	42.81
Granular(3)	56.38	77.48	73.31	73.31	74.61
Gravel(7)	47.83	48.89	46.65	46.65	47.88
Groats(7)	46.49	56.53	54.80	54.80	57.81
Leather(5)	57.58	78.60	72.27	72.27	72.27
Mineral(6)	46.16	56.64	54.75	54.75	56.71
Paper(10)	62.15	89.26	88.91	88.91	90.94
Pasta(6)	53.13	56.19	51.17	51.17	52.21
Pellet(4)	46.49	50.00	48.63	48.63	47.66
Plastic(47)	37.60	50.38	48.12	48.12	49.17
Quartz(6)	44.92	51.56	49.74	49.74	52.54
Rubber(1)	98.83	92.19	85.94	85.94	92.58
Sand(5)	46.95	45.47	44.77	44.77	46.56
Sandpaper(8)	42.77	42.53	41.85	41.85	43.75
Seeds(13)	58.00	58.17	55.83	55.83	57.87
Tile(7)	28.13	42.64	38.95	38.95	41.52
Wallpaper(20)	48.77	63.56	65.37	65.37	69.55
Wood(12)	41.34	66.02	70.64	70.64	72.98
Wool(2)	48.05	57.62	55.86	55.86	59.57
**Average**	**45.88%**	**52.21%**	**51.42%**	**51.42%**	**53.18%**

### 2.3 Extraction of Texture Features

#### 2.3.1 Extraction of texture features based on nonsubsampled contourlet transform

A nonsubsampled contourlet transform includes a nonsubsampled pyramid and nonsubsampled directional filter banks [13]–[14]. A nonsubsampled pyramid includes a set of two-channel nonsubsampled filter banks (NSFB). Nonsubsampled filtering does not implement a downsampling operation on an image but implements upsampling for filter banks, so NSCT has not only anisotropy but also the shift invariance.

Two–level NSCT decomposition is shown in **Figure 1**.

A nonsubsampled Laplace pyramid is a two-channel nonsubsampled transform. The condition of perfect reconstruction is shown as the following,
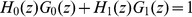
(6)Where, 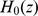 and 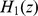 denote low frequency and high frequency decomposition filters. 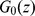 and 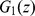 denote low frequency and high-frequency reconstruction filters. For practical image decomposition, a nonsubsampled àtrous wavelet transform is used to obtain a high frequency subband and a low frequency subband; a certain number of directional filters are then used to get some directional subbands. In order to get multiresolution analysis, one can continue to decompose the à trous wavelet transform low frequency subband.

Each image is decomposed five levels using a nonsubsampled Laplace pyramid decomposition; all the obtained high frequency subbands then continue to be decomposed by the nonsubsampled directional filter banks. A “pyr” pyramidal filter and a “vk” directional filter are used to lower the time complexity in our experiments, because they both have relatively small support. After the above decomposition process, 31 high frequency subbands and 1 low frequency subband are obtained. The mean 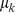 and the standard deviation 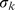 of the subband coefficients are calculated. There are 32 subbands: the feature vector 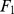 is constructed by 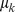 and 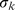 as the following,
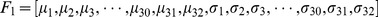
(7)


#### 2.3.2 Extraction of texture features based on a dual-Tree complex wavelet transform

DT-CWT is usually used to extract texture features in a wavelet domain. An image can be decomposed into two low frequency subbands and six high frequency subbands by DT-CWT in every level. The low frequency subbands can be decomposed again. In our experiments, an image is decomposed into three levels by DT-CWT. All together, there are two low frequency subbands and eighteen high frequency subbands. The mean 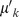 and the standard deviation 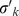 of the coefficients in each subband are calculated. There are 20 subbands; the feature vector 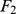 is constructed by 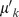 and 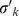 as the following,
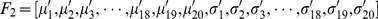
(8)


**Figure 5:**
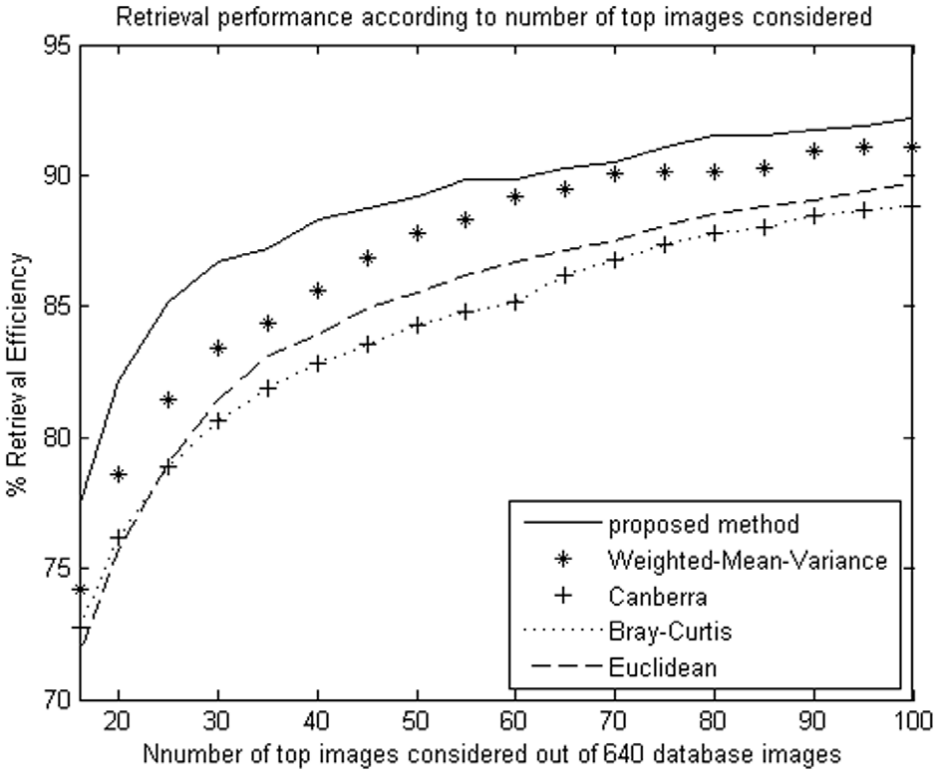
Average retrieval rate of database according to the number of top retrieved images using NSCT. The MIT image database (640 images) were used.

**Figure 6:**
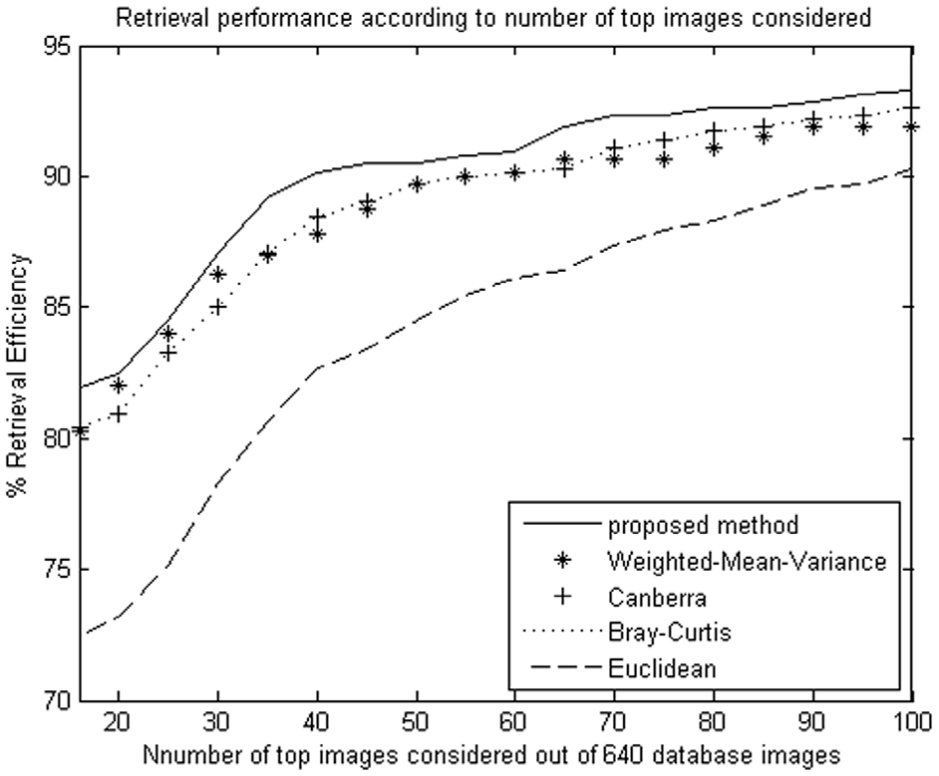
Average retrieval rate of database according to the number of top retrieved images using DT-CWT. The MIT image database (640 images) were used.

**Figure 7:**
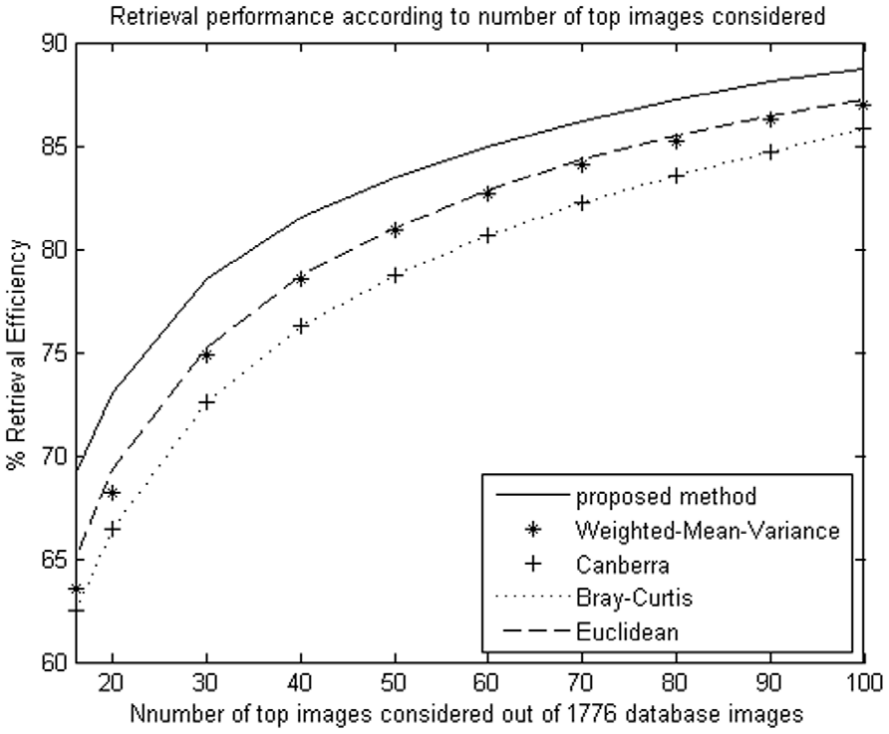
Average retrieval rate of database according to the number of top retrieved images using NSCT. The Brodatz image database (1776 images) were used.

**Figure 8:**
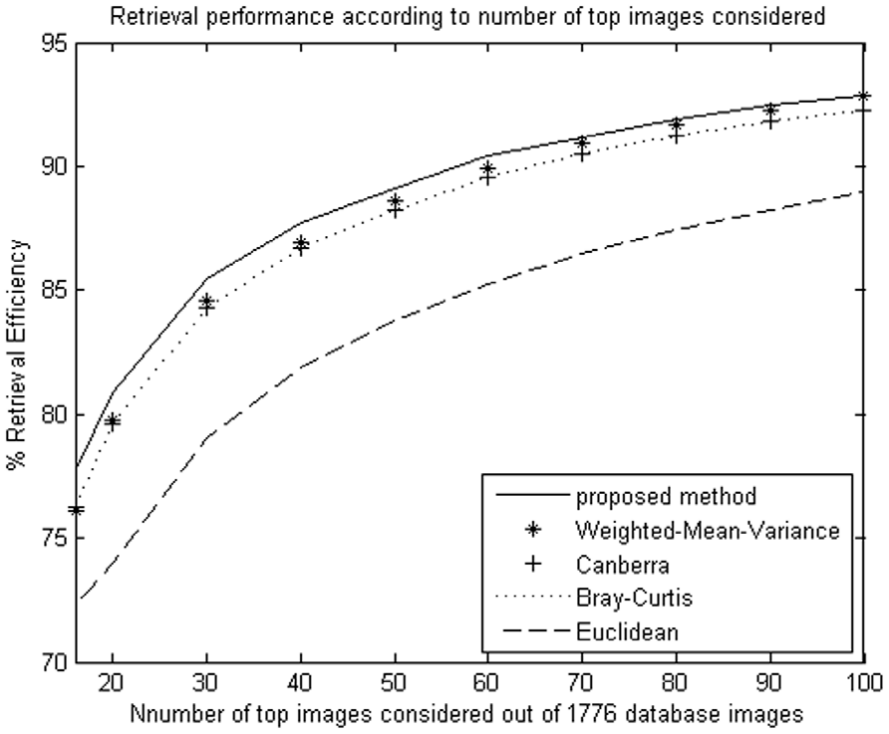
Average retrieval rate of database according to the number of top retrieved images using DT-CWT. The Brodatz image database (1776 images) were used.

**Figure 9:**
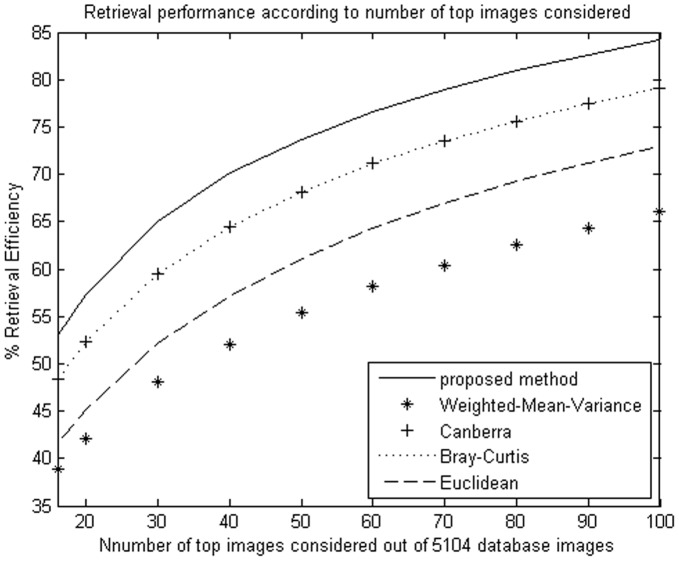
Average retrieval rate of database according to the number of top retrieved images using NSCT. The Outex image database (5104 images) were used.

**Figure 10:**
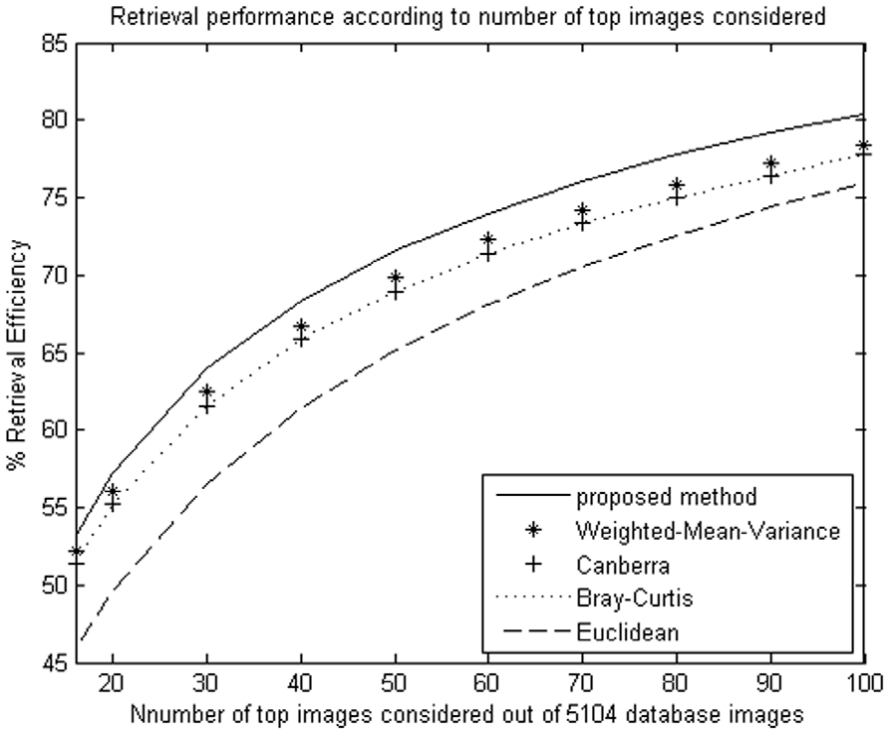
Average retrieval rate of database according to the number of top retrieved images using DT-CWT. The Outex image database (5104 images) were used.

## Results

### 3.1 Image Database

#### 1) MIT image database

In the retrieval experiments, real-world 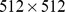 images of different natural scenes from the Massachusetts Institute of Technology (MIT) Vision Texture database are used [16]. There are 640 texture images from 40 different classes from the MIT texture database in the image database. Each original MIT texture image is divided into sixteen nonoverlapping 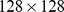 subimages. The total number of images in this database is 640 (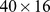). The query image is any one of the 640 subimages; the other 15 subimages from the same class are relevant candidate images. Forty different classes from the MIT texture database are shown as **Figure 2**.

#### 2) Brodatz image database

There are 1776 texture images from 111 different classes from the Brodatz texture database [17] in the image database. Each original Brodatz texture image is divided into sixteen nonoverlapping 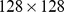 subimages. The total number of images in this database is 1776 (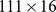). The query image is any one of the 1776 subimages; the other 15 subimages from the same class are relevant candidate images. Different classes from the Brodatz texture database are shown as **Figure 3**.

#### 3) Outex image database

There are 5104 texture images from 319 different classes from the Outex texture database [18] in the image database. Each original Outex texture image is divided into sixteen nonoverlapping 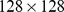 subimages. The total number of images in this database is 5104 (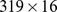). The query image is any one of the 5104 subimages; the other 15 subimages from the same class are relevant candidate images. Different classes from the Outex texture database are shown as **Figure 4**.

### 3.2 The Existing and the Proposed Texture Image Retrieval Methods

In our experiments, each image is decomposed by DT-CWT and NSCT respectively. Ten kinds of texture image retrieval methods are given as follows:

**Method 1**: use DT-CWT and ED.

**Method 2**: use DT-CWT and WMVD.

**Method 3**: use DT-CWT and CD.

**Method 4**: use DT-CWT and BCD.

**Method 5** : use DT-CWT and AED.

**Method 6**: use NSCT and ED.

**Method 7**: use NSCT and WMVD.

**Method 8**: use NSCT and CD.

**Method 9**: use NSCT and BCD.

**Method 10** : use NSCT and AED.

### 3.3 Experimental Results

The average precision ratio is used to evaluate retrieval performance. The average precision ratio is calculated using the following formula,
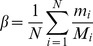
(9)Where 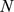 is the total number of the images in the texture database. 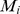 is the number of similar images that belong to the same class in the image database. 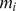 is the number of images that are properly ferreted out from the texture database in practice. In this paper, there are sixteen 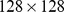 subimages in the same class. The number of top retrieved images is considered as 16. 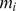 is the number of images of the top 16 retrieved images belonging to the same class.

The proposed method improves retrieval performance on the database, compared with the other four similarity measurements. The experimental results are shown as **Table 1**, **Table 2** **,** **Table 3**, **Table 4**, **Table 5** **, and** **Table 6**.

We evaluate the performance in terms of the average retrieval rate of relevant images as a function of the number of top retrieved images; the retrieval performance is shown as **Figure 5** **,** **Figure 6** **,** **Figure 7** **,** **Figure 8** **,** **Figure 9** **,** and **Figure 10**. The experimental results show that the proposed similarity measurement can improve average precision on the image retrieval.

## Discussion

The ED measurement lacks the interrelations between objects. When the ED measurement is used as a similarity measurement for texture image retrieval in a wavelet domain, retrieval accuracy is not always satisfied. The WMVD similarity measurement has been widely used for image retrieval, but it has a shortcoming: the similarity measurement between two images is sometimes affected by some uncorrelated images in the whole image database, because the standard deviation of all the images in the whole database needs to be calculated in order to normalize the Euclidean distance. CD and BCD similarity measurements use the difference and the normalization of the difference between two image features; they do not have scaling effects, but their drawback is that they both sum differences in a simple manner. The proposed similarity measurement first uses the denominator to normalize the difference between the two image features, so it can avoid scaling effects. Next, the differences in each dimension are squared, and they are summed before extracting the square root. The proposed similarity measurement can comprehensively use all features; it can avoid the limitations of CD and BCD similarity measurements.

### Conclusion

We present an effective similarity distance measurement for image retrieval. Features of all the images were extracted using DT-CWT and NSCT respectively. Experimental results demonstrate that the proposed similarity distance measurement achieves higher retrieval accuracy than some existing similarity measures.
